# *Sm*MYC2b Enhances Tanshinone Accumulation in *Salvia miltiorrhiza* by Activating Pathway Genes and Promoting Lateral Root Development

**DOI:** 10.3389/fpls.2020.559438

**Published:** 2020-09-11

**Authors:** Yangyun Zhou, Jingxian Feng, Qing Li, Doudou Huang, Xiao Chen, Zenan Du, Zongyou Lv, Ying Xiao, Yonglong Han, Junfeng Chen, Wansheng Chen

**Affiliations:** ^1^ Department of Pharmacy, Changzheng Hospital, Naval Medical University (Second Military Medical University), Shanghai, China; ^2^ Department of Pharmacy, Shanghai Jiao Tong University Affiliated Sixth People’s Hospital, Shanghai, China; ^3^ Research and Development Center of Chinese Medicine Resources and Biotechnology, Institute of Chinese Materia Medica, Shanghai University of Traditional Chinese Medicine, Shanghai, China

**Keywords:** *Salvia miltiorrhiza*, *Sm*MYC2b, tanshinone biosynthesis, lateral root development, transcriptome

## Abstract

*Salvia miltiorrhiza* Bunge (Lamiaceae) is an economically important medicinal plant as well as an emerging model plant. Our previous studies indicate that *Sm*MYC2b is a positive transcription factor that can affect the biosynthesis of phenolic acids and tanshinones in *S. miltiorrhiza.* Moreover, MYC2s are well known to induce the development of lateral roots. As tanshinones are mainly distributed in the periderm, the promotion of lateral root development probably leads to increased accumulation of tanshinones. In this paper, we firstly discovered that *Sm*MYC2b played a dual regulatory role in effectively enhancing the tanshinone accumulation by activating tanshinone biosynthetic pathway and promoting lateral root development. The expression levels of the previously studied pathway genes *SmCPS1*, *SmKSL1*, *SmCYP76AH1*, *SmCYP76AH3*, and *SmCYP76AK1* dramatically increased. In addition, *Sm*MYC2b was proved to exhibit a similar function as other homologs in promoting lateral root development, which increased the tanshinone produced tissue and further enhanced the biosynthesis of tanshinones. RNA-seq assays revealed that *Sm*MYC2b-regulated genes comprised 30.6% (1,901 of 6,210) of JA-responsive genes, confirming that *Sm*MYC2b played a crucial role in transcriptional regulation of JA-regulated genes. Overall, we concluded that *Sm*MYC2b could enhance tanshinone accumulation by activating the tanshinone biosynthetic pathway and promoting lateral root development. Our study provides an effective approach to enhance the production of desired tanshinones and enriches our knowledge of the related regulatory network.

## Introduction

*Salvia miltiorrhiza* Bunge (Lamiaceae) is a perennial herb that is a source of the famous traditional Chinese medicine (TCM) Danshen. Cultivated in East Asia, the dried roots and rhizomes of *S. miltiorrhiza* are mainly used in treating coronary heart disease, angina, acute ischemic stroke, and other cardiovascular diseases (Lin and Hsieh, 2010). The bioactive components in *S. miltiorrhiza* are mainly lipophilic diterpenoids (including tanshinone IIA, tanshinone I, tanshinone IIB, and cryptotanshinone) and hydrophilic phenolics (such as salvianolic acids and rosmarinic acid) (Gong et al., 2011; Su et al., 2015). Based on previous studies, the biosynthesis of terpenoids consists of three stages: isoprene precursor biosynthesis, direct skeleton precursor generation, and the biosynthesis of various tanshinones (Chen et al., 2011; Chang et al., 2019; Xue et al., 2019). The first stage mainly generates isopentenyl pyrophosphate (IPP) and dimethylallyl pyrophosphate (DMAPP) derived from the mevalonate (MVA) pathway and the methylerythritol phosphate (MEP) pathway (Vranová et al., 2013; Zhao et al., 2013). The second stage is the generation of farnesyl diphosphate (FPP), geranyl diphosphate (GPP) and geranylgeranyl diphosphate (GGPP), which further form the precursors of monoterpenes, sesquiterpenes, and diterpenes respectively (Liang et al., 2002; Szkopińska and Płochocka, 2005). At present, the first two stages have been studied clearly and are shared by all terpenes. The third stage involves a variety of terpenoid synthases and modifying enzymes, which determine the structural diversity of terpenoids and are species specific (Keeling et al., 2008; Degenhardt et al., 2009).

The metabolic engineering of one or more rate-limiting enzymes is an effective strategy to increase the metabolic flux towards the desirable compounds. Significant progress has been made in increasing the production of the bioactive compounds by overexpression of the key enzymes (Shi et al., 2016; Shi et al., 2020; Zhang et al., 2020). A large number of bioactive compounds are distributed in the specific tissues, such as the antitumor drug paclitaxel and the antimalarial drug quinine, which are mainly distributed in the bark. To reach high yield of the bioactive components, increasing the producing tissues can be an ideal strategy. The β-glucosidase offers a possibility to increase artemisinin accumulation by promoting gland density in *Artemisia annua* (Singh et al., 2015).

Tanshinone pigments accumulate in the periderm (Xu et al., 2015), while salvianolic acid B (Sal B) is mainly detected in the phloem and xylem of roots (Xu et al., 2016b). As tanshinones are mainly distributed in the periderm, the rhizomes/roots of *S. miltiorrhiza* are typically reddish brown. This is also consistent with the classification and screening of TCM Danshen. During the screening of Danshen materials, roots with different morphologies are often used in four applications. It is generally accepted that the tanshinone content is higher in the dark reddish brown and deeply branched *S. miltiorrhiza* roots. This kind of material is generally used to extract tanshinones. Phenolic acids are usually extracted from coarser roots. Materials with good shape and pharmacopoeia standard content are used for slicing. Others are used in the extraction of tanshinones and phenolic acids together.

It will be exciting to realize the dual activation of the tanshinone biosynthetic pathway and the specific tissue development. The biosynthesis of the tanshinones and the development of specific tissues are regulated by many factors, among which transcription factors (TFs) that control the abundance or activity of multiple enzymes have become promising regulators (Zhang et al., 2015; Deng et al., 2020a). In our previous studies, the TAR1 TF is demonstrated to play a dual regulatory role in the artemisinin biosynthesis by controlling trichome development and promoting artemisinin pathway in *A. annua* (Tan et al., 2015). In *S. miltiorrhiza*, it is not clear whether there is an ideal TF with similar dual function. The MYC2 TF is widely known to induce the formation of lateral roots (Yadav, 2005; Sun et al., 2009). As tanshinones are mainly distributed in the periderm, overexpressing *MYC2b* may promote the development of lateral roots to enhance the content of tanshinones. Our previous studies indicate that *Sm*MYC2b is a positive JA-responsive TF that facilitates the biosynthesis of tanshinone IIA and salvianolic acid B (Zhou et al., 2016). Tanshinones and phenolic acids may also accumulate following overexpressing of *MYC2b*. Overexpressing *MYC2b* could be a potential strategy to increase tanshinones in one step by promoting tanshinone pathway gene expression and lateral root development.

In the present paper, we implemented transcriptome profiling by RNA sequencing (RNA-seq) to mine MYC2-targeted genes. We showed that *Sm*MYC2b could promote the biosynthesis of tanshinone biosynthesis genes (*SmCPS1*, *SmKSL1*, *SmCYP76AH1*, *SmCYP76AH3*, and *SmCYP76AK1*). *Sm*MYC2b mainly modulated the downstream tanshinone pathway, instead of the nonspecific upstream pathway. The content of tanshinones dramatically increased with the exceptional decrease in carnosic acid (CA), suggesting its role as an important tanshinone precursor. MYC2b was also proposed to positively regulate lateral root development. These data suggest that *Sm*MYC2b dramatically enhances the accumulation of tanshinones by activating the tanshinone pathway and promoting lateral root development in *Salvia miltiorrhiza.*

## Materials and Methods

### Plant Materials and Growth Conditions

Danshen (*Salvia miltiorrhiza* Bunge) plants were cultivated in the botanical garden of the Second Military Medical University, Shanghai, China. To obtain sterile *S. miltiorrhiza* seedlings, seeds were immersed in 75% alcohol for 1 min and afterwards were washed three times with distilled water. After subsequent treatment with 0.1% HgCl_2_ for 5 min, the seeds were rinsed three times for 3 min each with sterile distilled water and incubated on Murashige and Skoog (MS) solid medium plus 3% sucrose with an appropriate pH value of 5.6. The sterilized seedlings were grown in a growth room at 24 ± 2°C under a 16-h light/8-h dark photoperiod for propagation.

Seeds of an *Arabidopsis thaliana* (L.) mutant were sown in a soil matrix. PCR identified approximately 1-month-old mutant plants with good growth status that were transformed by the soaking method. The aboveground parts of the *A. thaliana* plants were immersed in transformation buffer for 5–15 s to ensure that all buds were immersed. Then, the plants were laid flat and covered to keep out light. The next day, the cover was removed, and the plants were raised and transferred to normal conditions for growth. Their seeds were collected and stored.

*S. miltiorrhiza* Bunge plants were also cultivated in the botanical garden of the Second Military Medical University, Shanghai, China. To examine the expression patterns of *SmMYC2b*, we collected roots of *S. miltiorrhiza* plants at different times (5, 15, 30, 60, 90, 120, 150, 180, 210, 240, 270, and 300 d). There were three biological replicates for every time point.

### Vector Construction and Plant Transformation

A modified *pHB-flag* vector that could express C-terminally Flag-tagged fusion protein was chosen as an overexpression vector. The full-length open reading frame (ORF) of *SmMYC2b* was amplified with primers 5’-TGATCAATGGGGGTTGTTGGTTGG-3’ and 5’-ACTAGTCCCGAGAGATAACTGATG-3’, and inserted into the *BamH* I and *Spe* I sites of the *pHB-flag* vector. The constructed *pHB-flag-MYC2b* vector was introduced into the disarmed *Agrobacterium tumefaciens* EHA105 strain. The *pHB-flag* vector was also transformed with the same method to serve as a control.

*Agrobacterium*-mediated construction of transgenic plants was performed according to previous protocols (Yan and Wang, 2007). Generally, 1-month-old *S. miltiorrhiza* plants with seven to eight leaves were chosen for genetic transformation. The leaves were cut into 0.5 × 0.5 cm discs. All explants were cocultured on MS basal medium with *A. tumefaciens* EHA105 at 25°C for 2 d in the dark. Afterwards, the explants were transformed to selective media supplemented with different concentrations of cefotaxime (from 500 mg/L gradually reduced to 0) and hygromycin (2 mg/L). The identification of MYC2b transgenic seedlings was conducted by western blot (WB) assay. Total soluble protein was extracted by adding Plant Protein Extraction Reagent (CWBIO, Beijing, China) supplemented with Protease Inhibitor Cocktail. The samples were denatured for 5 min in boiling water with protein loading buffer. After separation on an 8% SDS-PAGE mini-gel, the targeted proteins were blotted to a nitrocellulose membrane. The blots were probed with the anti-FLAG mouse antibody (1:1,000, Sigma). The corresponding HRP-labelled goat anti-mouse IgG(H+L) antibody (Beyotime, China) was used as a secondary antibody at a final dilution of 1:1,000. A Chemiscope 3000 mini was used to capture chemiluminescence according to the manufacturer’s instructions (Clinx, Shanghai, China). The fresh roots from five selected lines were subjected to root imaging analysis as biological replicates. The roots were weighed and collected for root imaging analysis using WinRHIZO software (WinRhizo, Regent Instruments, Canada).

### Changes in Root Morphological Caused by *Sm*MYC2b in *A. thaliana*

The constructed *pHB-flag-MYC2b* vector was introduced into *A. thaliana* MYC2 mutant (*jin1-9*) lines to generate complementary mutant *SmMYC2b* lines. Different genotypes of *A. thaliana* seeds (Col-0, *jin1-9*) were sown on 0.5×MS plates for germination. The *SmMYC2b* seeds were sown on hygromycin B selective medium. Then, after 7 d, the seedlings were plated on 0.5×MS with 50 μM methyl jasmonate (Sigma) or a solvent control on square plates and were finally transferred to a growth room in a vertical position. Root growth was monitored regularly, plates were photographed and root length was measured after 7 d of vertical cultivation.

### MeJA Treatment and Construction of RNA-Seq Libraries

Two-month-old CK and *MYC2bOE* sterile seedlings grown on 0.5×MS medium were treated with either 100 μM MeJA or mock solution (ethanol). After treatment for 1 h, roots from the treated seedlings were collected from three biological replicates. Total RNA was extracted using an RNeasy Plant Mini Kit (Qiagen, German) following the user manual. The extracted RNA was quantified using a NanoDrop 2000c spectrophotometer (Thermo Fisher Scientific, America) and assessed using an Agilent 2100 Bioanalyser (Agilent Technologies, America). Total RNA (3 μg for each sample) was used to construct mRNA libraries according to the manufacturer’s instructions. Sequencing was conducted on an Illumina HiSeq 2500 platform.

### Analysis of RNA-Seq Data

The *S. miltiorrhiza* reference genome and corresponding gene model annotation files were downloaded directly from public genome websites (Xu et al., 2016a). More than three biological replicates (six MYC2bOE lines, four CK lines, three MeJA-treated MYC2bOE lines, and three MeJA-treated CK lines) were sequenced on an Illumina HiSeq 2500. Bowtie 2 v2.1.0 (Langmead and Salzberg, 2012) was used to map the clean reads from each sample to reference sequence transcripts. The differentially expressed genes (DEGs) were screened using the DEGseq v1.20.0 (Wang et al., 2010) package with the MARS (MA-plot-based method with random sampling model) model. When the following conditions were all satisfied: |log_2_Fold change| ≥ 1, FDR (q value) < 0.001 and RPKM of at least one sample >20, the genes were considered differentially expressed. To clarify differences in gene function, gene ontology (GO) enrichment analysis was implemented using GOseq (Young et al., 2010) based on the Wallenius noncentral hypergeometric distribution. GO terms with corrected P-values <0.05 were considered significantly enriched in the DEGs. The GO terms with FDR value (q value) <0.05 were considered strikingly enriched in the DEGs.

### Quantitative Real-Time PCR (qRT-PCR)

The relative expression analysis of *S. miltiorrhiza* genes was verified by qRT-PCR. Total RNA was isolated using a commercial kit (TIANGEN, Beijing, China). One microgram of the extracted total RNA was reverse transcribed to generate first strand cDNA using a TransScript^®^ First-Strand cDNA Synthesis SuperMix Kit (TransGen Biotech, Beijing, China). qRT-PCR assays were conducted on a Termal Cycler Dice Real Time System TP800 according to the manufacturer’s instructions (Takara Bio Inc., Dalian, China). Expression levels were normalized to the corresponding housekeeping gene (18S RNA gene). All qRT-PCR experiments were carried out with three independent replicates. Gene-specific primers were designed and are listed in **Table S1**.

### Chromatin Immunoprecipitation Assay

Chromatin immunoprecipitation (ChIP) assays were conducted according to previously published protocols (Nelson et al., 2006; Abdelaty et al., 2008). Briefly, 1 g of fresh root tissues was collected and cross-linked using 1% formaldehyde under vacuum for 15 min. The reaction was stopped by adding glycine to a final concentration of 0.125 M. Then, the roots were ground to a fine powder in liquid nitrogen. The chromatin was extracted, sonicated, and immunoprecipitated with ChIP grade rabbit anti-FLAG antibodies (CST #14793S). The protein-DNA complex was reverse cross-linked, and the protein was digested. Subsequently, the ChIP-DNA was purified and subjected to ChIP-qPCR. The gene-specific primers are listed in **Table S2**.

### Compound Extraction and Analysis

Positive MYC2b*OE* seedlings were confirmed by western blot assay. The roots of six transgenic and four independent control seedlings were subjected to compound analysis. The harvested roots were dried at 40°C overnight to a constant dry weight and milled to homogeneous powders that could sieve through No. 100 mesh. Active compounds containing lipophilic tanshinones and hydrophilic phenolic acids were extracted following previously reported methods (Zhu et al., 2007; Gu et al., 2012). A 0.02 g powder sample was precisely weighed and extracted in 4 ml 70% methanol under sonication for 30 min. After centrifugation at 10,000 rpm for 5 min, the supernatant was collected and filtered through a 0.22 μm organic membrane. Afterwards, the samples were injected into the HPLC system for content analysis by applying the standard curve method. TT was designated as the summed content of DTI, TI, CT, and TIIA.

### CA Treatment Assay

Based on the above research, a proposed biosynthetic route from CA to tanshinones was presumed. Wild-type hairy roots of *S. miltiorrhiza* from the same line were grown in specialized MSOH liquid culture. Plants with uniform growth were randomly divided into two groups: the CA treatment group and the control group. CA dissolved in 70% ethanol was added into the liquid medium to reach a final concentration of 50 μg/ml, while controls were treated with 70% ethanol. The hairy roots were harvested for tanshinone content determination after treatment for 0, 1, 2, 4, and 8 d. The collected hairy roots were dried at 50°C and the determination was consistent with the above method. The experiment was performed with three independent replicates.

### Data Availability

The raw RNA-seq read data are publicly available at the NCBI Sequence Read Archive with the accession number PRJNA623221 (http://www.ncbi.nlm.nih.gov/sra/).

## Results

### Overexpression of *Sm*MYC2b in Transgenic *S. miltiorrhiza* Plants Causes Morphological Changes

**Figure 1:**
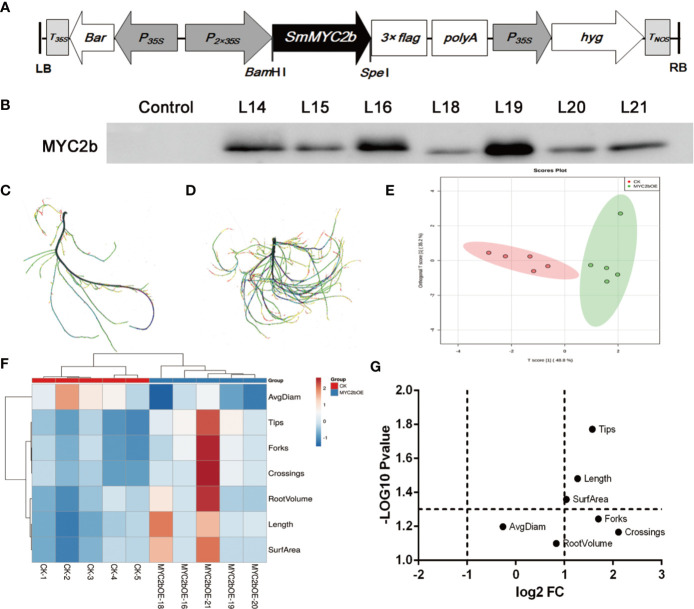
*Sm*MYC2b promotes the development of lateral roots in *S. miltiorrhiza.* **(A)** Schematic diagram of the constructed plant overexpression vector *pHB-flag-MYC2b*. **(B)** Western blot analysis of the *Sm*MYC2b protein in transgenic *S. miltiorrhiza* plants. L14, L15, L16, L18, L19, L20, and L21 denote different MYC2b overexpression lines. **(C, D)** show CK and *MYC2bOE* sterilized plants, respectively, with different root morphological characteristics. **(E)** PCA analysis, **(F)** heatmap, and **(G)** volcano plot of roots from CK and *SmMYC2b* transgenic plants. The analysis was performed with five independent replicates.

To further investigate the regulatory effect of *Sm*MYC2b on active compounds, we constructed the *SmMYC2b* overexpression vector *pHB-flag-MYC2* (**Figure 1A**) to generate transgenic plants. The growth process of the transgenic plants is shown in **Figure S1**. Positive *MYC2b* overexpression (*MYC2bOE*) plants were identified using the WB method (**Figure 1B**). Compared with CK, we surprisingly found that *MYC2bOE* plants exhibited a much more developed root systems (**Figures 1C, D**). Notably, obvious lateral root phenotypes were observed in *MYC2bOE* plants. Then, WinRHIZO software was applied to capture root morphological trait data including length, surface area (SurfArea), root volume (RootVolume), average diameter (AvgDiam), tips, forks, and crossings. Principal component analysis (PCA) was applied as a powerful tool to reduce the complexity of the data. As shown in **Figure 1E**, the OPLS-DA plot denoted that the samples could be classified into two groups, MYC2bOE and CK. A heatmap (**Figure 1F**) was generated and coupled with hierarchical clustering analysis to further understand the difference in root characteristics among the transgenic and CK plants. The X-axis shows the clustering of the two groups, including MYC2bOE and CK. Root tips, forks, and crossings on the Y-axis were clustered into one group as they were correlated with each other. RootVolume, length, and SurfArea were correlated with each other. Volcano plots are a powerful tool to screen different root traits between two groups and display two important indicators, fold change (FC) and p-value, in one image. The root length, tips, and SurfArea were differential root indexes based on their fold changes and P-values (**Figure 1G**). The forks and crossings of MYC2bOE were more than 2-fold those of CK. However, the results of the t-test did not show a significant difference due to the excessive intragroup differences.

### *Sm*MYC2b Rescues the Phenotype of the *A. thaliana* MYC2 Mutant

**Figure 2:**
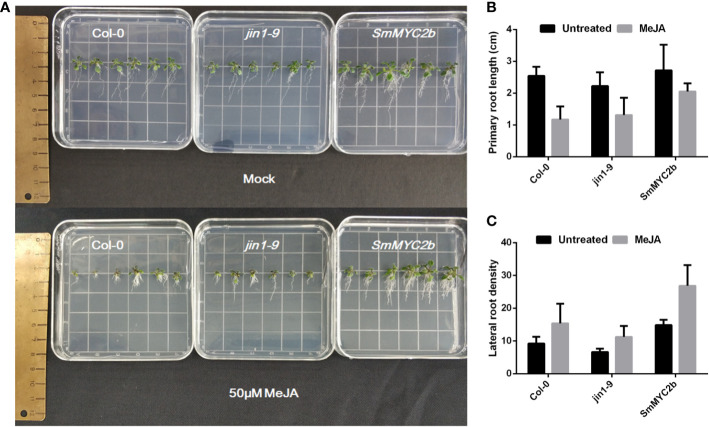
MYC2b facilitates the development of lateral roots without inhibition of primary roots. **(A)** Photographs of Col-0 (wild type), *jin1-9* (MYC2 mutant type), and *SmMYC2b* (*SmMYC2b* overexpressed in *jin1-9*) *A. thaliana* plants with or without MeJA treatment. **(B)** Primary root length of different groups in **(A)**; Values are the means with SE (n = 6). **(C)** Lateral root density of different groups in **(A)**. Lateral root density was given as the number of lateral roots calculated per centimeter of primary root. Values are the means with SE (n = 6).

Previous studies indicate that MYC2 positively promoted lateral root formation and inhibited primary root elongation. To investigate whether *Sm*MYC2b exhibited similar functions in roots, transgenic plants were produced. Different genotypes of *A. thaliana* seeds (Col-0, *jin1-9* and *SmMYC2b*) were harvested, sown, and treated with 100 μM MeJA or not treated (mock group). As shown in **Figure 2**, the roots with different genotypes or treatments exhibited different phenotypes. Two-way ANOVA with genotype and treatment as factors was performed to examine significant differences between groups. Genotype (wild type versus *jin1-9 versus* *SmMYC2b)* and treatment (mock *versus* MeJA treatment) caused differences in primary root length and lateral root density, respectively (**Table S3**). Moreover, there was no interaction between the two factors. Overall, JA was discovered to promote the development of lateral roots and reduce the elongation of primary roots. Unlike JA, *Sm*MYC2b dramatically promoted lateral root formation without inhibiting the primary root elongation.

### *Sm*MYC2b Promotes the Biosynthesis of Tanshinones

**Figure 3:**
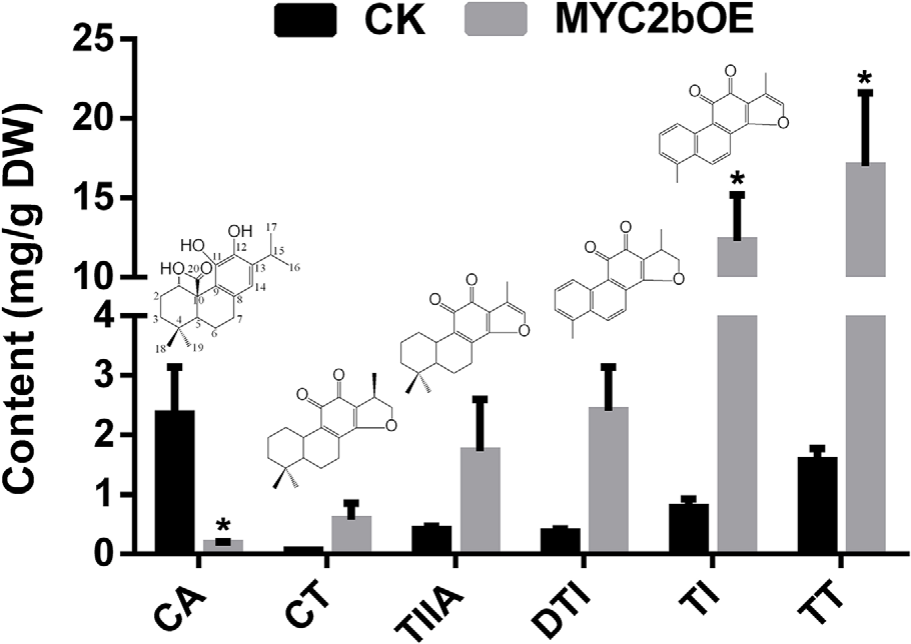
Overexpression of *SmMYC2b* enhances the biosynthesis of tanshinones. Values are the means with SE (n = 3–4). Asterisks suggest significant differences at P < 0.05; CA, carnosic acid; DTI, dihydrotanshinone I; TI, tanshinone I; CT, cryptotanshinone; TIIA, tanshinone IIA; TT, total tanshinone.

To investigate the effect of *Sm*MYC2b on phenolic acids and tanshinones, we determined the active compounds of transgenic *MYC2bOE* and CK plants. Among the six determined MYC2b overexpression lines, three lines that exhibited extremely significant enhancement in tanshinone content were subjected to content analysis. A significant increase in tanshinones (15.74-fold TI and 10.89-fold TT) is shown in **Figure 3**. The DTI, CT, and TIIA contents respectively increased 5.63-, 8.01-, and 3.13-fold, respectively, in OE compared with CK plants. However, t-test results did not exhibit significant differences owing to excessive intragroup differences. *MYC2bOE-19* contained 9.46-fold DTI, 15.40-fold CT, and 8.22-fold TIIA contents compared with those of the CK. However, the other *MYC2bOE* lines harbored much higher contents. In addition, the content of CA, which was proposed to be an upstream compound of tanshinones according to its molecular structure, dramatically decreased. There was no obvious change in the content of the compounds (Phe, Tyr, CinA, 4-HPPA, DSS, HGA, CafA, RA, SAA, and SAB) in the SAB pathway (**Figure S2**). ChIP-qPCR assays were conducted to validate the RNA-seq results. *Sm*CPS1 and *Sm*KSL1, which are involved in the tanshinone pathway, were discovered to be *Sm*MYC2b-regulated genes whose promoter sequences were downloaded from the GenBank database with the accession numbers KY937191 and KY937192, respectively (**Figure S3**).

Tanshinones predominantly accumulated in the roots of *S. miltiorrhiza*, especially in the rhizome. To provide more evidence supporting the physiological roles of *SmMYC2b* in tanshinone biosynthesis, we examined the expression pattern of *SmMYC2b*. In general, the expression level of *SmMYC2b* increased with time (**Figure S4**).

### *Sm*MYC2b Positively Regulates Tanshinone Biosynthesis Genes to Enhance the Biosynthesis of Tanshinones

**Figure 4:**
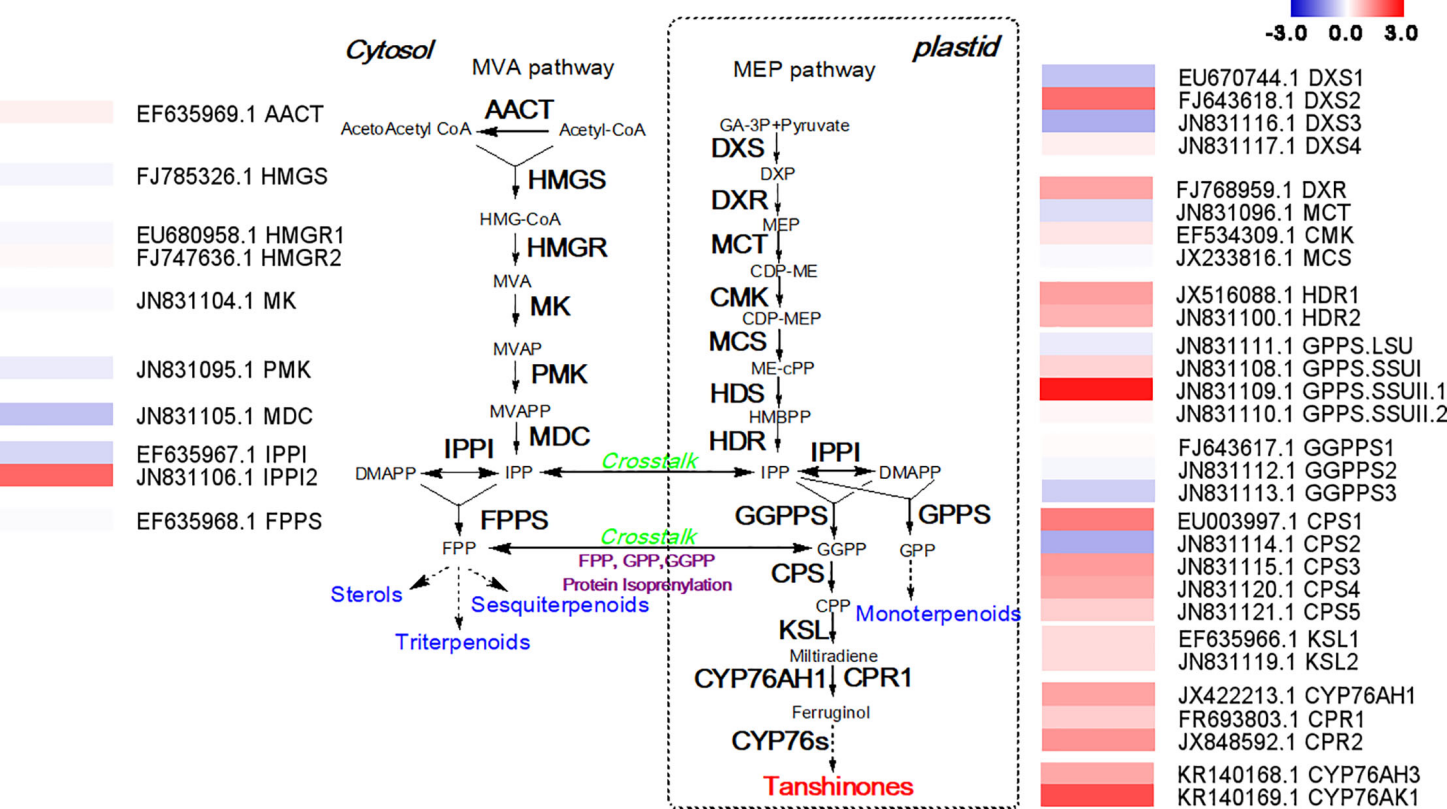
Overexpression of *SmMYC2b* promotes the expression of tanshinone biosynthetic genes. AACT, acetoacetyl-CoA thiolase; CDP-ME, 4-(cytidine 5’-diphospho)-2-C-methyl- D-erythritol; CDP-MEP, 2-phospho-4-(cytidine 5’-diphospho)-2-C-methyl-D-erythritol; CMK, 4-diphosphocytidyl-2C-methyl-D-erythritol kinase; CPP, copalyl diphosphate; Cprimary root, cytochrome p450 reductase; CPS, copalyl diphosphate synthase; CYP, cytochrome p450; DMAPP, dimethylallyl pyrophosphate; DXP, 1-deoxy-D-xylulose 5-phosphate; DXR, 1-deoxy-D-xylulose 5- phosphate reductoisomerase; DXS, 1-deoxy-D-xylulose-5-phosphate synthase; FPP, farnesyldiphosphate; FPPS, farnesyl diphosphate synthase; GA-3P, glyceraldehyde-3-phosphate; GGPP, geranylgeranyl diphosphate; GGPP, geranylgeranyl diphosphate; GGPPS, geranylgeranyl diphosphate synthase; GPP, geranyl diphosphate; GPPS, geranyl diphosphate synthase; HDR, 1-hydroxy-2-methyl-2-(E)-butenyl 4-diphosphate reductase; HDS, 1-hydroxy-2-methyl-2-(E)-butenyl 4-diphosphate synthase; HMBPP, 4-hydroxy-3-methylbut-2-enyl diphosphate; HMG-CoA, 3-hydroxy-3-methylglutaryl-coenzyme A; HMGR, 3-hydroxy-3-methylglutaryl-CoA reductase; HMGS, 3-hydroxy-3-methylglutaryl-CoA synthase; IDS, isoprenyl diphosphate synthase; IPP, isopentenyl pyrophosphate; IPPI, isopentenyl diphosphate isomerase; KSL, kaurene synthase-like; MCS, 2C-methyl-D-erythritol 2,4-cyclodiphosphate synthase; MCT, 2C-methyl-D-erythritol 4-phosphate cytidyl transferase; MDC, mevalonate diphosphate decarboxylase; ME-cPP, 2-C-methyl-D-erythritol-2,4-cyclodiphosphate; MEP, 2-C-methyl-d-erythritol 4-phosphate; MK, Mevalonate kinase; MVA, mevalonate; MVAP, mevalonate 5-phosphate; MVAPP, mevalonate 5-diphosphate; PMK, phosphomevalonate kinase. The fold change of gene expression levels measured in RPKM is shown in a log2 scale. Red represents upregulated genes and blue indicates downregulated genes.

Because the mechanism by which how MYC2b affects the biosynthesis of tanshinones is unclear, RNA-seq analysis was conducted to mine potential tanshinone biosynthesis genes. The tanshinones were derived from the cytosol-localized mevalonic acid (MVA) pathway and plastid-localized methylerythritol phosphate (MEP) pathway. The expression levels of genes involved in the MVA pathway were almost unchanged (**Figure 4**). There was no obvious change in upstream genes in the MEP pathway. However, downstream genes in the MEP pathway significantly increased, suggesting that MYC2b mainly targets relative downstream biosynthesis genes to increase the content of tanshinones. *SmCPS1* and *SmCPS2* were proposed to be involved in distinct tanshinone pathways in the root periderm and aerial tissues respectively. The expression level of *SmCPS1* was discovered to be increased while that of *SmCPS2* decreased, collectively leading to an increase in the tanshinone content. *SmKSL1* was discovered to be quite specific in its production of miltiradiene from CPP. CYP76AH1 was able to efficiently catalyze the conversion of miltiradiene to ferruginol in the tanshinone pathway. CYP76AH3 and CYP76AK1 formed a bifurcating pathway for the biosynthesis of tanshinones. The increase in the expression levels of *SmCPS1*, *SmKSL1*, *SmCYP76AH1*, *SmCYP76AH3*, and *SmCYP76AK1* collectively enhanced tanshinone production. The expression levels of genes in the SAB pathway did not exhibit significant changes, which is consistent with the content determination results (**Figure S5**).

### Transcriptome Analysis of MYC2b-Regulated Genes During JA Signaling

**Figure 5:**
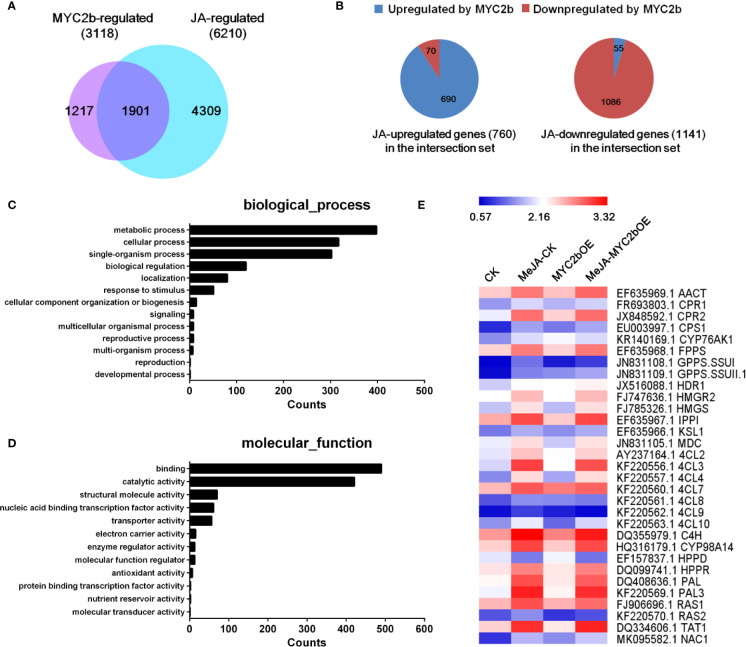
*Sm*MYC2b plays a vital role in the JA pathway. **(A)** Venn diagram illustrating the overlap of JA-regulated genes and MYC2b-regulated genes. **(B)** Distribution of MYC2b-upregulated and MYC2b-downregulated genes among the 1901 JA and MYC2b co-regulated genes. **(C, D)** GO analysis of JA and MYC2b co-regulated genes, respectively. **(E)** Expression of typical genes regulated by JA. The average fragments per kilobase of exon per million fragments mapped (RPKM, log10 scale) of each gene is shown.

Based on its efficient and extensive regulatory characteristics, MYC2 is considered a regulatory hub within the JA signaling pathway (Kazan and Manners, 2012; Du et al., 2017). The role of MYC2 orthologues in the regulation of JA-mediated biosynthesis of secondary metabolites has been well established. To investigate the role of MYC2b in the JA pathway and screen MYC2b-regulated genes, we performed RNA-seq analysis. Transcriptome profiles of four groups including CK and MYC2bOE plants with or without MeJA treatment, were compared by a pairwise comparison method to screen MYC2b-regulated genes (MYC2bOE *vs* CK), JA-regulated genes (MeJA-CK *vs* CK) and interacting genes (shown in **Dataset 1**). We identified 6,210 JA-regulated genes, 3,118 MYC2b-regulated genes, and 1,901 interacting genes (**Figure 5**). GO analysis of JA and MYC2b co-regulated genes was performed and is shown in **Figures 5C, D**. The co-regulated genes were mainly involved in metabolic processes, cellular processes, single-organism processes, biological regulation, and localization. The majority of these genes exerted binding and catalytic functions. JA-regulated genes and MYC2b-regulated genes exhibited similar gene functions (**Figure S6**). JA-regulated tanshinone and SAB pathway genes are also displayed in **Figure 5E**. As the group of JA-regulated genes included MYC2b-regulated genes and non-MYC2b-regulated genes, 4,309 non-MYC2b-regulated genes were discovered. To further narrow MYC2b-regulated genes, we compared DEGs of MeJA-MYC2bOE vs CK plants with MeJA-CK vs CK plants. The considerably upregulated or downregulated genes were designated MYC2b-regulated genes (2,018). The number of interacting genes between the 2,018 MYC2b-regulated genes and the above 3,118 MYC2b-regulated genes was narrowed to 666 (shown in **Dataset 2**). Sixteen lateral root development genes regulated by *Sm*MYC2b are listed in **Table S4**.

### CA Was Presumed to Be an Important Precursor of Tanshinones

**Figure 6:**
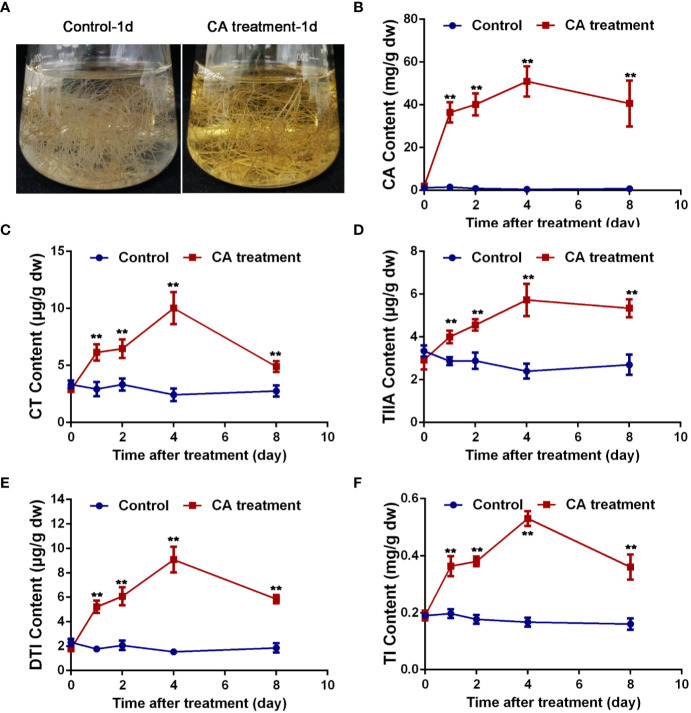
Exogenous CA treatment caused changes in tanshinone accumulation. **(A)** The hairy root medium treated with CA for 1 d turned yellow. **(B–F)** exhibited content changes in CA, CT, TIIA, DTI, and TI, respectively. Asterisks denote significant differences at P < 0.05. Values are the means with SE (n = 3).

Based on the above results, CA was presumed to be a potential precursor of tanshinones. To further explain the relationship between CA and tanshinones, CA feeding studies were carried out. *S. miltiorrhiza* hairy root cultures were used as materials, which were characterized by a fast growth rate and were genetically stable (Guillon et al., 2006; Miklaszewska et al., 2017). As shown in **Figure 6**, the color of the liquid medium in which *S. miltiorrhiza* hairy roots were growing surprisingly turned yellow after treatment for 1 d, faded slowly after 4 d, and returned to transparent after 8 d. At the same time, the content of CA, CT, TIIA, DTI, and TI increased significantly after CA treatment, reached the maximum values at 4 d and then decreasing. On the 4th day, the content of CA, CT, TIIA, DTI, and TI reached 95.50, 4.12, 2.39, 5.90, and 3.26 times those of the control, respectively. The exogenous addition of CA greatly increased the content of CA, CT, TIIA, DTI, and TI, suggesting that CA was probably an important precursor of tanshinone components. More direct evidence should be provided to explain the relationship between CA and tanshinones.

## Discussion

To improve plant survival, plant terpenoids serve as phytoalexins that can effectively resist pest and pathogen attack (Schmelz et al., 2011; Schmelz et al., 2014). Previous reports indicate that the terpenoid contents change during plant development in many plant species (Niu et al., 2015; Aizpurua-Olaizola et al., 2016). Thus, to characterize the secondary metabolism in plants, plant growth and development should be taken into consideration. MYC2 was discovered to be an efficient regulator in the modulation of secondary metabolites. However, there is still limited understanding of the regulatory effects of *Sm*MYC2b on bioactive compounds. To investigate the expression level of *Sm*MYC2b throughout the plant life cycle, we collected *S. miltiorrhiza* roots at various time points. Overall, the expression level of MYC2b increases with plant aging.

Lateral roots help anchor the plant securely in the soil as well as engage in water and nutrient uptake. In the present study, *Sm*MYC2b promoted lateral root development. This result is consistent with previous findings in other species (Yadav, 2005). A previous investigation demonstrates that JA represses the expression of PLT1 and PLT2 transcription factors to inhibit primary root elongation, and this effect is executed by MYC2 (Shen et al., 2016; Ishimaru et al., 2018). However, *Sm*MYC2b exerts opposite functions in regulating primary root growth. *Sm*MYC2b stimulates root growth by promoting lateral root development and primary root elongation. As tanshinone pigments are mainly distributed in the periderm (Xu et al., 2015), the promotion of root development is accompanied by an increase in tanshinone producing tissues, leading to enhanced accumulation of tanshinones.

The plant hormone JA plays noteworthy roles in multiple plant processes, including plant resistance, growth, and development. MYC2 is known as a vitally important TF involved in most aspects of the JA signaling pathway (Kazan and Manners, 2012; Du et al., 2017). Here, RNA-seq assays revealed that *Sm*MYC2b-regulated genes comprised 30.6% (1,901 of 6,210) of JA-responsive genes. Moreover, the interacting genes (the MYC2b and JA co-regulated genes) comprised 60.9% (1,901 of 3,118) of the MYC2b-regulated genes. As *Sm*MYC2a was formerly designated as a similar TF of *Sm*MYC2b (Zhou et al., 2016), *Sm*MYC2a and *Sm*MYC2b are proposed to exert the most influence on JA function.

The metabolic engineering of key TFs provides a feasible strategy for improving the content of tanshinones and other bioactive compounds (Sun et al., 2019; Deng et al., 2020b; Hao et al., 2020). As MYC2 orthologs are master regulators of JA-mediated secondary metabolite biosynthesis, metabolic engineering using *Sm*MYC2b as candidate can be a promising strategy to achieve high yield of target compounds in *S. miltiorrhiza*. Previous knockdown of *Sm*MYC2b causes a significant decrease in tanshinone and SAB biosynthesis, indicating that *Sm*MYC2b is a positive regulator performing similar functions as other MYC2 homologs (Dombrecht et al., 2007; Zhang et al., 2011; Shen et al., 2016). More efforts should be made to explain the related complicated regulatory networks. In this study, RNA-seq data were sufficiently analyzed to mine clues supporting the above deduction. *Sm*MYC2b specifically enhances the expression of genes involved in the downstream pathway of tanshinones, including *SmCPS1*, *SmKSL1*, *SmKSL2*, *SmCYP76AH1*, *SmCYP76AH3*, and *SmCYP76AK1*. Among the characterized *SmCPS1-5* genes, only *SmCPS1* exhibits catalytic activity in tanshinone biosynthesis in the roots (Cui et al., 2015). *SmKSL1* was discovered to specifically catalyze the next reaction step in the production of miltiradiene (Gao et al., 2009). Miltiradiene can be effectively converted into ferruginol by *SmCYP76AH1* (Guo et al., 2013). *SmCYP76AH3* and *SmCYP76AK1* both exhibit promiscuity, leading to a diversity of tanshinones (Guo et al., 2016).

Overexpression of *SmMYC2b* also leads to activation of the tanshinone pathway. Compared with CK, the contents of DTI, TI, CT, and TIIA exhibited different degrees of increase. In contrast, the content of CA dramatically decreased. By far, the tanshinone biosynthetic pathway hasn’t been completely elucidated, extremely the specific downstream pathway. The latest work on *S. miltiorrhiza* characterized *CYP76AH3* which catalyzes ferruginol to 11-hydroxy ferruginol (Guo et al., 2016). In other species including *Rosmarinus officinalis*, *Salvia fruticose*, and *Salvia pomifera*, 11-hydroxyferruginol is converted to CA by CYP76AK6-8 (Ignea et al., 2016; Scheler et al., 2016). In *S. miltiorrhiza*, whether CYP76AK6-8 homologs are involved in similar processes has not yet been reported. CA belongs to the group of labdane-type diterpene, and is synthesized from geranylgeranyl diphosphate (GGPP) (Ignea et al., 2016; Scheler et al., 2016). Based on the structural characteristics, it is reasonable to deduce that CA can be converted to CT by decarboxylation reaction and cyclization reaction. Afterwards, the metabolic flux is proposed to occur successively from CT, TIIA, DTI, to TI by dehydrogenation reactions. Coincidentally, the contents of CT, TIIA, DTI, and TI were discovered to successively increase, leading to the intensive accumulation of terminal products. However, the content of CA dramatically decreased. As the expression levels of CA upstream pathway genes, including *SmCPS1*, *SmKSL1*, *SmCYP76AH1*, and *SmCYP76AH3*, consistently increase, CA accumulation should increase, in contrast with the content determination results. It is reasonable to deduce that the decrease in CA content leads to an increase in tanshinones, indicating that CA is a crucial precursor of tanshinones. However, more evidence is required to support this hypothesis.

To further explore the function of CA, CA feeding studies were carried out. After treatment for 4 d, the contents of CA, CT, TIIA, DTI, and TI reached their maximum values. The contents of CA, CT, TIIA, DTI, and TI reached 95.50, 4.12, 2.39, 5.90, and 3.26 times those of the control, respectively. The exogenous addition of CA greatly increased the contents of CA, CT, TIIA, DTI, and TI, suggesting that CA is an essential precursor of tanshinone components that provide raw material for the biosynthesis and regulation of tanshinone components. MYC2b promotes the transformation of the CA precursor to tanshinones to realize extremely high accumulation of end products. Moreover, *Sm*MYC2b mainly modulates the downstream genes of the tanshinone pathway instead of the upstream genes, which are shared with other pathways, such as monoterpene and sesquiterpene pathways. Our previous studies indicate that *Sm*MYC2b is a positive transcription factor that can affect the biosynthesis of phenolic acids and tanshinones in *S. miltiorrhiza* hairy roots. Phenolic acids and tanshinones are supposed to be increased by overexpression of *Sm*MYC2b. However, there was no significant up-regulating of SAB pathway, suggesting the role of endogenous *Sm*MYC2b is sufficient for the induction of SAB biosynthesis genes (Zhang et al., 2011).

The extensive roles of JA in the regulation of pathway genes involved in bioactive compounds are well established (Wasternack and Hause, 2013). MYC2 is a master regulator that modulates wide range of functions triggered by JA signaling pathway (Kazan and Manners, 2012; Du et al., 2017; Wasternack and Song, 2017). As JA-induced biosynthesis of many secondary metabolites is executed by MYC2 (Zhou and Memelink, 2016), overexpression of *Sm*MYC2b can be a promising method to increase the tanshinone yield in *S. miltiorrhiza*. In this paper, *Sm*MYC2b is proved to promote two physiological aspects of *S. miltiorrhiza* roots, tanshinone biosynthesis and lateral roots development, which are both decisive factors of yield and quality of this medicinal material. Thus, *Sm*MYC2 might provide an efficient regulatory target for breeding of *S. miltiorrhiza*, as well as a basis for exploring the mechanism behind the extensive roles of MYC2 in planta.

## Data Availability Statement

The raw RNA-seq read data are publicly available at the NCBI Sequence Read Archive with the accession number PRJNA623221 (http://www.ncbi.nlm.nih.gov/sra/).

## Author Contributions

YZ, JC, and WC planned and designed the research. YZ, JF, QL, DH, XC, ZD, JC, ZL, YX performed experiments. YZ, JF, DH, YH, JC, and WC analyzed data. YZ and JC wrote the manuscript. All authors contributed to the article and approved the submitted version.

## Funding

We are grateful to Shujuan Zhao and Ronghui Tan for preparing materials. This work was financially supported by the National Natural Science Foundation of China (81603220, 81673550, 31970325, and 31770329), National Key R&D Program of China (2019YFC1711100) and Shanghai Rising-Star Program (18QB1402700, China).

## Conflict of Interest

The authors declare that the research was conducted in the absence of any commercial or financial relationships that could be construed as a potential conflict of interest.
