# Supplementary figures and images for: SmMYC2b Enhances Tanshinone Accumulation in Salvia miltiorrhiza by Activating Pathway Genes and Promoting Lateral Root Development
Source: Front Plant Sci. 2020 Sep 11;11:559438. doi: 10.3389/fpls.2020.559438 (PMC7517298; doi:10.3389/fpls.2020.559438)

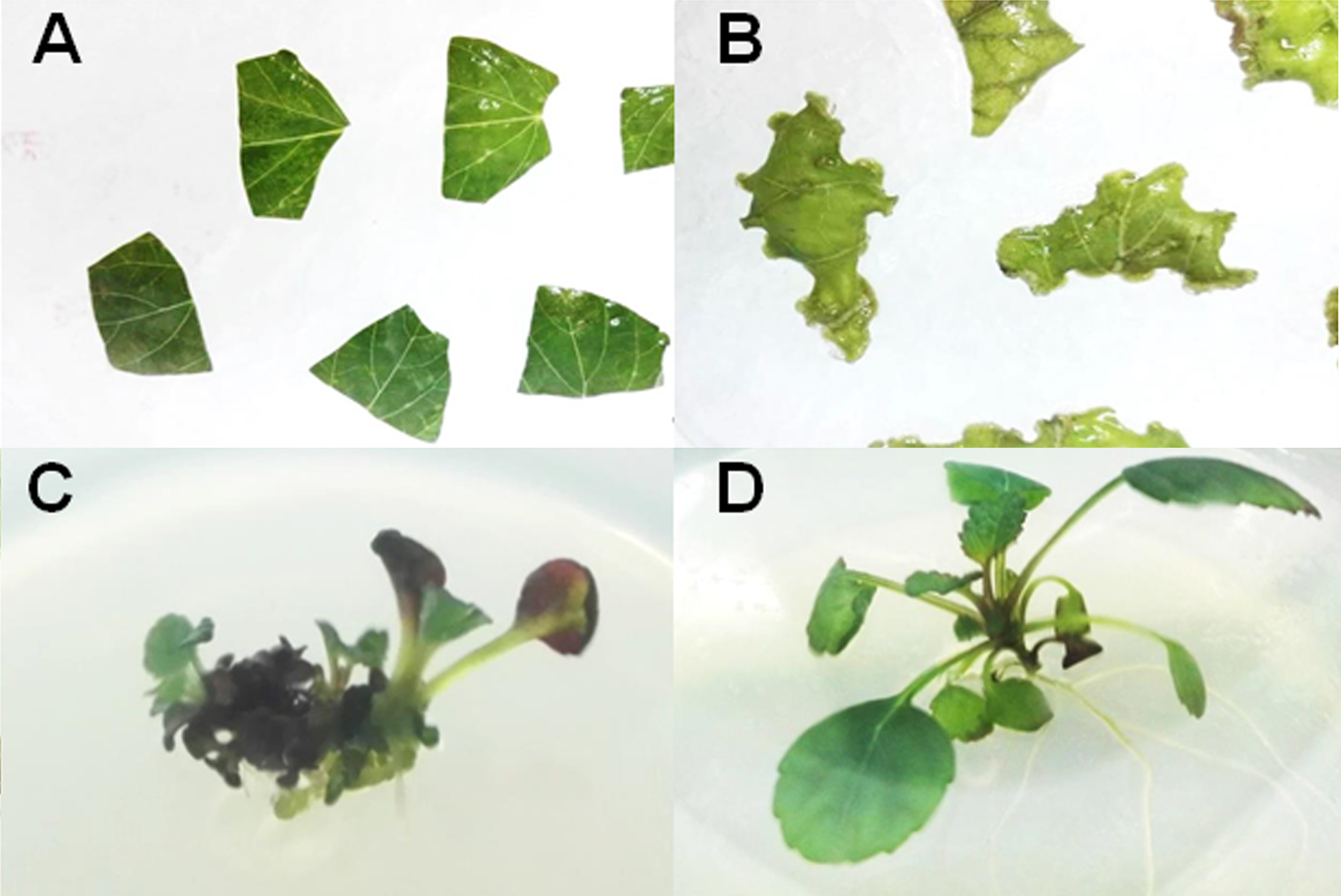

Supplement: Supplementary file 1 [file Image_1.tif]

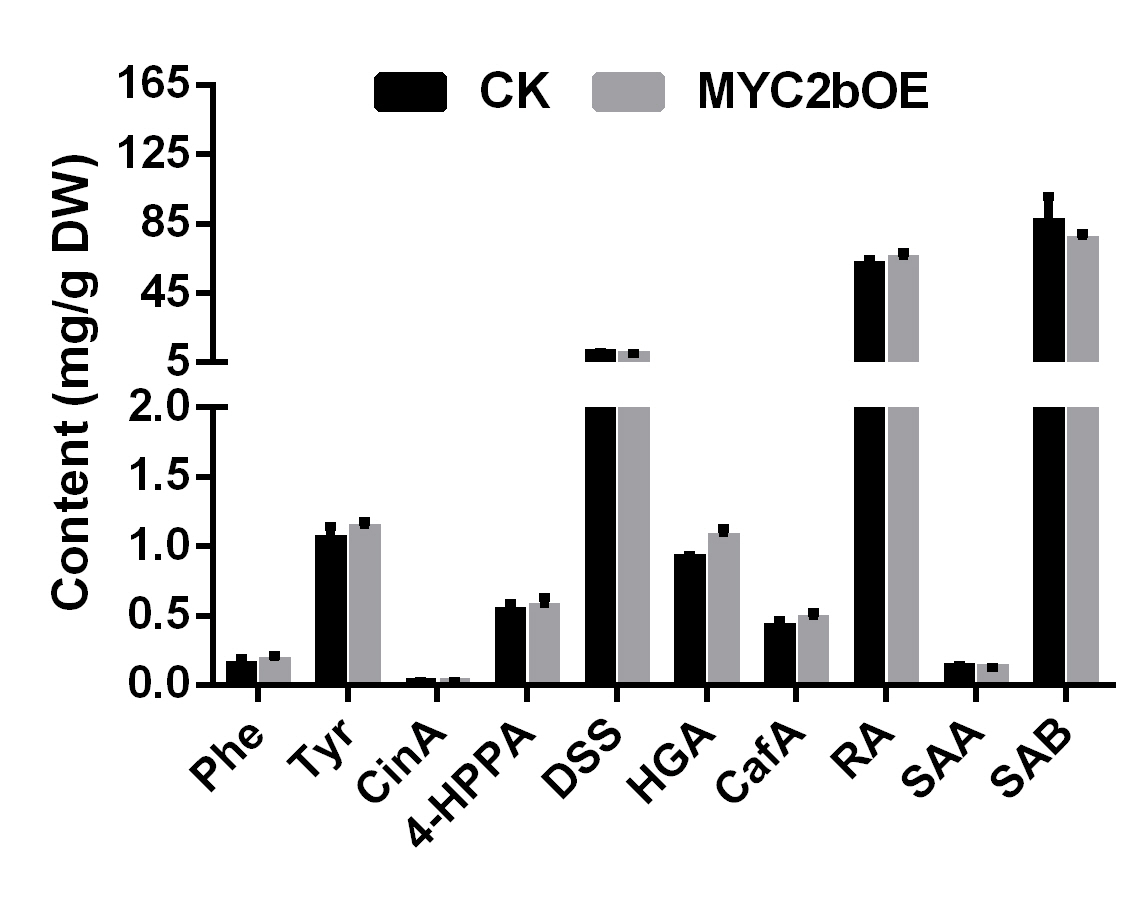

Supplement: Supplementary file 2 [file Image_2.tif]

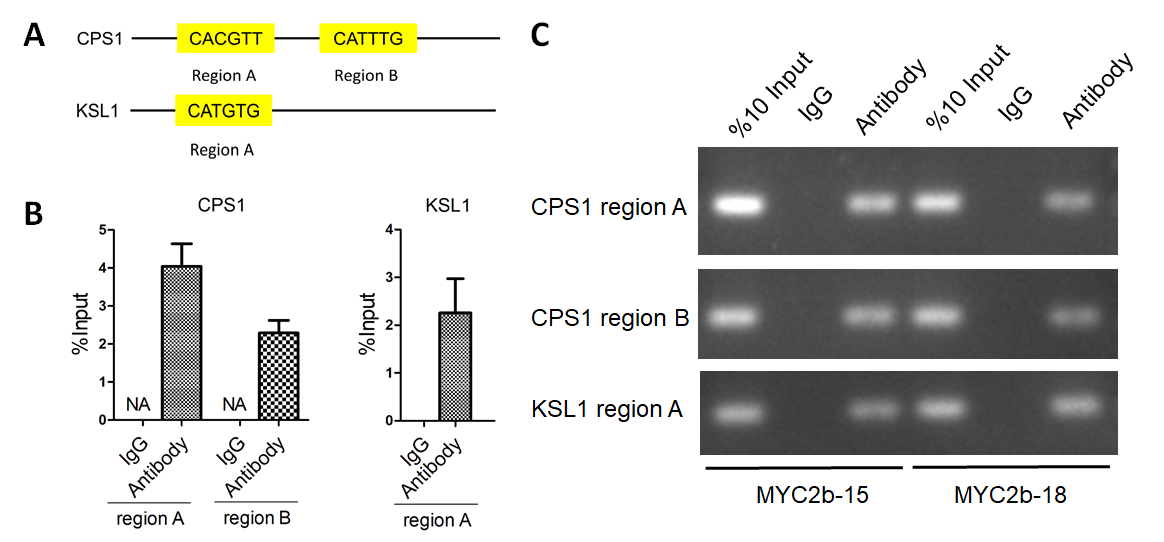

Supplement: Supplementary file 3 [file Image_3.tif]

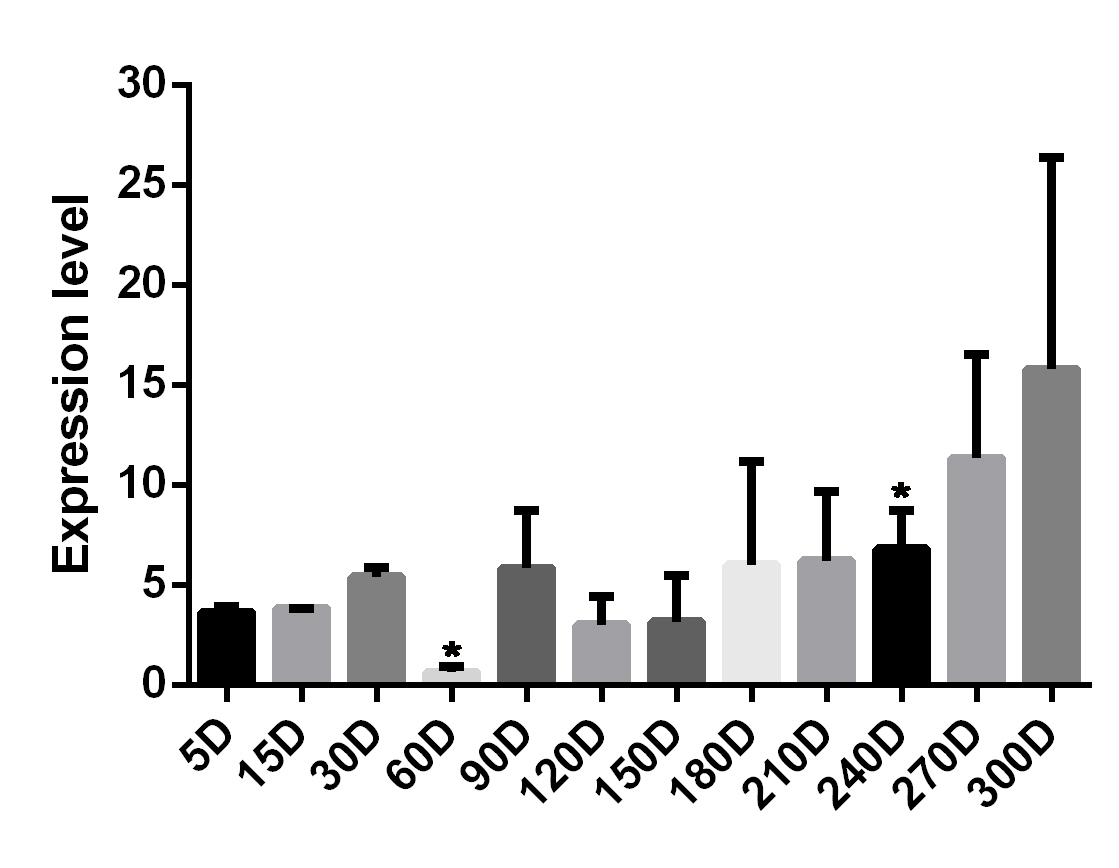

Supplement: Supplementary file 4 [file Image_4.tif]

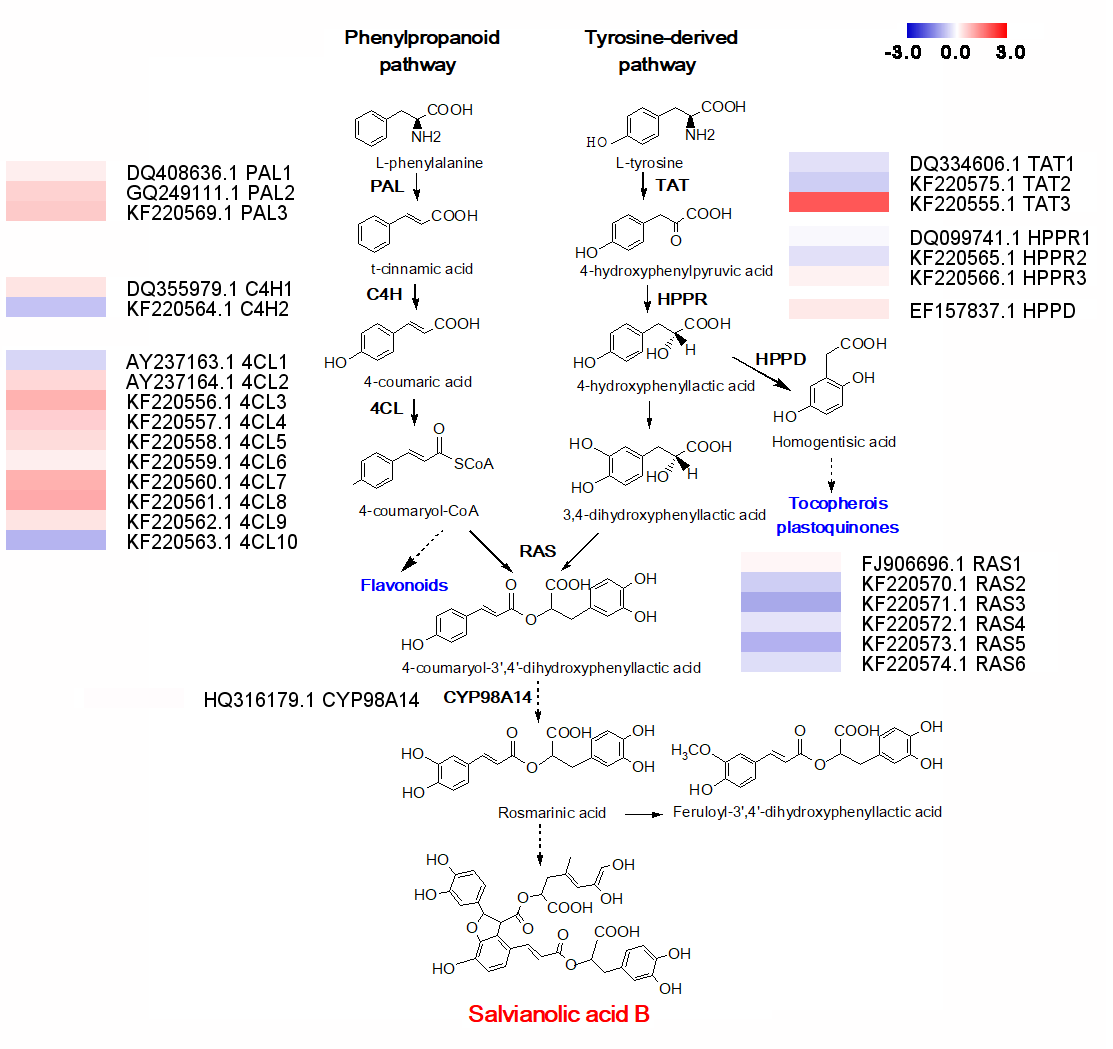

Supplement: Supplementary file 5 [file Image_5.tif]

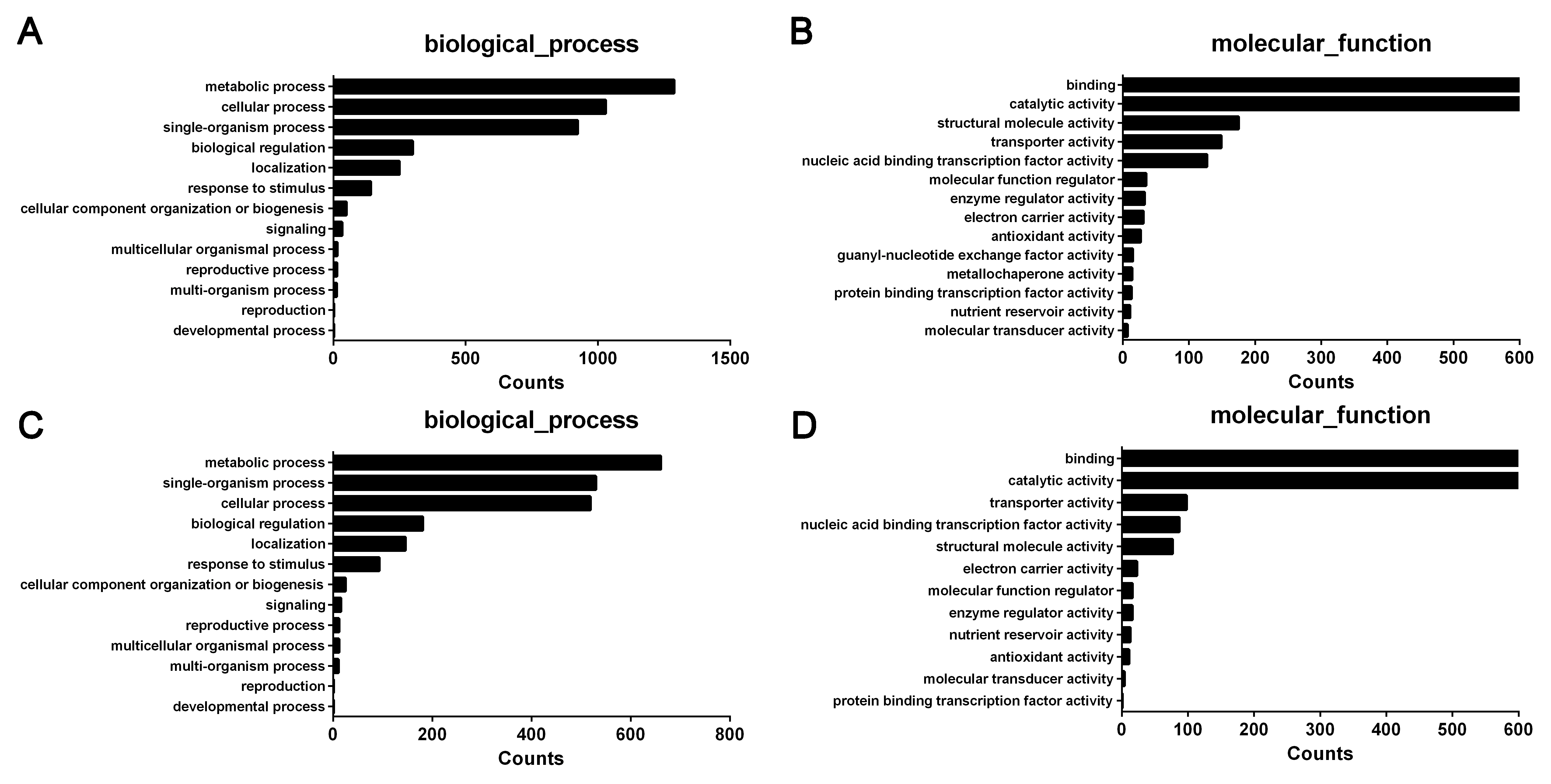

Supplement: Supplementary file 6 [file Image_6.tif]
