# Supplementary material for: SmMYC2b Enhances Tanshinone Accumulation in Salvia miltiorrhiza by Activating Pathway Genes and Promoting Lateral Root Development
Source: Front Plant Sci. 2020 Sep 11;11:559438. doi: 10.3389/fpls.2020.559438 (PMC7517298; doi:10.3389/fpls.2020.559438)
Supplement: Supplementary file 7 [file Table_1.docx]

Table S1 Primers of *Sm*MYC2s and pathway genes used for qRT-PCR analysis.

| Primer name | | Sequence (5'→3') | Note |  |
| --- | --- | --- | --- | --- |
| *SmMYC2b-*RTF | | GTTGCCAACGGGAATAGAG | | qRT-PCR(*Sm*MYC2b) |
| *SmMYC2b-*RTR | | GCTTCCTCGGCCTCTTATC | |  |
| *SmCMK-*RTF | | CCTTGGTGGTGGCAGCAGTAA | | qRT-PCR(*SmCMK*) |
| *SmCMK-*RTR | | GGTGGAGGAATATCTTCGACG | |  |
| *SmGGPPS1-*RTF | | TGAAATCACGGAAGCGCATT | | qRT-PCR (*SmGGPPS1*) |
| *SmGGPPS1-*RTR | | TCGTGGATCATCGGTGGATT | |  |
| *SmCPS1-*RTF | | ACTACCGTTCATCAAGGCCA | | qRT-PCR (*SmCPS*) |
| *SmCPS1-*RTR | | CCTCGAGTTGATTCTGCACG | |  |
| *SmKSL1-*RTF | | AGAGGGCTCATGTCGAACAA | | qRT-PCR (*SmKSL1*) |
| *SmKSL1-*RTR | | TTCTGCAGCCAATTGACACC | |  |
| *SmCYP76AH1-*RTF | | TCGTGGATGAGTCGGCAAT | | qRT-PCR (*SmCYP76AH1*) |
| *SmCYP76AH1-*RTR | | TGAGTATCTGAGTTCCCT | |  |
| *SmCYP76AH3-*RTF | | AACCCCACGAGACATTC | | qRT-PCR (*SmCYP76AH3*) |
| *SmCYP76AH3-*RTR | | CCGAGATGGACCGACA | |  |
| *SmCYP76AK1-*RTR | | CTACTCCACCCCGACAA | | qRT-PCR (*SmCYP76AK1*) |
| *SmCYP76AK1-*RTR | | CGGATTCCTCCACGAT | |  |
| *SmPAL1-*RTF | | ACCTACCTCGTCGCCCTATGC | | qRT-PCR (*SmPAL1*) |
| *SmPAL1-*RTR | | CCACGCGGATCAAGTCCTTCT | |  |
| *SmC4H1-*RTF | | CCAGGAGTCCAAATAACAGAGCC | | qRT-PCR (*SmC4H1*) |
| *SmC4H1-*RTR | | GAGCCACCAAGCGTTCACCAA | |  |
| *Sm4CL1-*RTF | | ATTCGCATTCGCATTTCTCGG | | qRT-PCR (*Sm4CL1*) |
| *Sm4CL1-*RTR | | GCGGCGTAGTGCTTCACCTTT | |  |
| *SmTAT1-*RTF | | TTCAACGGCTACGCTCCAACT | | qRT-PCR (*SmTAT1*) |
| *SmTAT1-*RTF | | AAACGGACAATGCTATCTCAAT | |  |
| *SmHPPR1-*RTF | | GACTCCAGAAACAACCCACATT | | qRT-PCR (*SmHPPR1*) |
| *SmHPPR1-*RTR | | CCCAGACGACCCTCCACAAGA | |  |
| *SmRAS6-*RTF | | CCCTCCATTTCATCAGCACG | | qRT-PCR (*SmRAS6*) |
| *SmRAS6-*RTR | | GATTTGTCGGTGTTGGGGAG | |  |
| *SmCYP98A14-*RTF | | CCATCATCGCCCTTCTTTGG | | qRT-PCR (*SmCYP98A14*) |
| *SmCYP98A14-*RTR | | TGTCCACTTCCGTCATCACA | |  |
| *Smactin-F* | | ATGATAACTCGACGGATCGC | | qRT-PCR (Housekeeping gene) |
| *Smactin-R* | | CTTGGATGTGGTAGCCGTTT | |  |
