# Supplementary material for: SmMYC2b Enhances Tanshinone Accumulation in Salvia miltiorrhiza by Activating Pathway Genes and Promoting Lateral Root Development
Source: Front Plant Sci. 2020 Sep 11;11:559438. doi: 10.3389/fpls.2020.559438 (PMC7517298; doi:10.3389/fpls.2020.559438)
Supplement: Supplementary file 8 [file Table_2.docx]

Table S2 Two-way ANOVA to examine significant differences between groups

|  | PR length | LR density |
| --- | --- | --- |
| MYC2b | 0.0091 | <0.0001 |
| MeJA | < 0.0001 | <0.0001 |
| Interaction | 0.2182 | 0.0721 |
