# Supplementary material for: SmMYC2b Enhances Tanshinone Accumulation in Salvia miltiorrhiza by Activating Pathway Genes and Promoting Lateral Root Development
Source: Front Plant Sci. 2020 Sep 11;11:559438. doi: 10.3389/fpls.2020.559438 (PMC7517298; doi:10.3389/fpls.2020.559438)
Supplement: Supplementary file 9 [file Table_3.docx]

Table S3 Primers used for ChIP-qPCR

| Gene | Sequence |
| --- | --- |
| Actin F | AGGAACCACCGATCCAGACA |
| Actin R | GGTGCCCTGAGGTCCTGTT |
| CPS1 region A F | CCAACCAAACTACCGATTTCAG |
| CPS1 region A R | TTCAATTTGGCTCGGAATCC |
| CPS1 region B F | GCCGCCTGTCCAATATTTTATAG |
| CPS1 region B R | CACTAATAATATTCTCCT |
| KSL1 region A F | GGTAGAAATTAGGGAAAT |
| KSL1 region A R | GTCATTCAATGAAAGACT |
