# Supplementary material for: SmMYC2b Enhances Tanshinone Accumulation in Salvia miltiorrhiza by Activating Pathway Genes and Promoting Lateral Root Development
Source: Front Plant Sci. 2020 Sep 11;11:559438. doi: 10.3389/fpls.2020.559438 (PMC7517298; doi:10.3389/fpls.2020.559438)
Supplement: Supplementary file 10 [file Table_4.docx]

Table S4 lateral root development genes regulated by *Sm*MYC2b

|  | **#Gene** | **Length** | **log2(Fold change)** | **p value** | **q value(BH adjust)** | **Result** | **Function** |
| --- | --- | --- | --- | --- | --- | --- | --- |
| 1 | SMil_00002987 | 873 | -1.899 | 0 | 0 | down | homeobox-leucine zipper protein HAT4-like |
| 2 | SMil_00025932 | 2661 | -1.787 | 1.4E-130 | 1.5E-129 | down | probable linoleate 9S-lipoxygenase 5 isoform X1 |
| 3 | SMil_00003799 | 1218 | 1.686 | 7.06E-71 | 4.68E-70 | up | patatin-like protein 1 |
| 4 | SMil_00029723 | 1203 | 1.553 | 7.89E-90 | 6.28E-89 | up | lon protease homolog 2, peroxisomal |
| 5 | SMil_00020906 | 3318 | 1.455 | 0 | 0 | up | auxin response factor ARF22 |
| 6 | SMil_00002879 | 927 | 1.452 | 1.96E-54 | 1.08E-53 | up | NAC domain-containing protein 100 |
| 7 | SMil_00002559 | 960 | 1.305 | 2E-175 | 2.8E-174 | up | NAC domain-containing protein 79 |
| 8 | SMil_00001334 | 3276 | 1.184 | 1.2E-163 | 1.6E-162 | up | auxin response factor ARF2 |
| 9 | SMil_00005248 | 1767 | 1.170 | 3.1E-214 | 5.2E-213 | up | serine/threonine-protein kinase Nek6 |
| 10 | SMil_00024652 | 8160 | 1.104 | 0 | 0 | up | LOW QUALITY PROTEIN: auxin transport protein BIG |
| 11 | SMil_00013175 | 2394 | 1.082 | 6.6E-145 | 7.8E-144 | up | WD repeat-containing protein 48 |
| 12 | SMil_00013392 | 954 | 1.068 | 0 | 0 | up | MYB-related transcription factor |
| 13 | SMil_00025172 | 3366 | 1.059 | 3.41E-76 | 2.39E-75 | up | protein SPA1-RELATED 2 |
| 14 | SMil_00012368 | 1383 | 1.045 | 4.3E-130 | 4.6E-129 | up | MYB-related transcription factor |
| 15 | SMil_00005319 | 942 | -1.043 | 0 | 0 | down | AP2/ERF and B3 domain-containing transcription factor RAV1-like |
| 16 | SMil_00001305 | 1089 | 1.001 | 0 | 0 | up | F-box protein SKP2A-like |
